# Higher admission rates and in-hospital mortality for acute type A aortic dissection during Influenza season: a single center experience

**DOI:** 10.1038/s41598-020-61717-5

**Published:** 2020-03-13

**Authors:** Carmel Ashur, Elizabeth Norton, Linda Farhat, Anna Conlon, Cristen Willer, James B. Froehlich, David J. Pinsky, Karen M. Kim, Shinichi Fukuhara, Michael G. Deeb, Himanshu Patel, Kim A. Eagle, Bo Yang, Marion A. Hofmann Bowman

**Affiliations:** 1 Department of Internal Medicine, Michigan Medicine, University of Michigan, Ann Arbor, MI USA; 2 Creighton University School of Medicine, Omaha, NE USA; 3 Frankel Cardiovascular Center, Michigan Medicine, University of Michigan, Ann Arbor, MI USA; 4 University of Michigan School of Medicine, Ann Arbor, MI USA

**Keywords:** Cardiology, Risk factors

## Abstract

Triggering events for acute aortic dissections are incompletely understood. We sought to investigate whether there is an association between admission for acute type A aortic dissection (ATAAD) to the University of Michigan Medical Center and the reported annual influenza activity by the Michigan Department of Health and Human Services. From 1996–2019 we had 758 patients admitted for ATAAD with 3.1 admissions per month during November-March and 2.5 admissions per month during April-October (p = 0.01). Influenza reporting data by the Michigan Department of Health and Human Services became available in 2009. ATAAD admissions for the period 2009–2019 (n = 455) were 4.8 cases/month during peak influenza months compared to 3.5 cases/month during non-peak influenza months (p = 0.001). ATAAD patients admitted during influenza season had increased in-hospital mortality (11.0% vs. 5.8%, p = 0.024) and increased 30-day mortality (9.7 vs. 5.4%, p = 0.048). The results point to higher admission rates for ATAAD during months with above average influenza rates. Future studies need to investigate whether influenza virus infection affects susceptibility for aortic dissection, and whether this risk can be attenuated with the annual influenza vaccine in this patient population.

## Introduction

Aortic dissection is a life-threatening condition which occurs when there is a tear in the wall of the aorta which allows blood to enter through the tear causing the aortic layers to separate or “dissect”. When the tear develops in the ascending aorta it is classified as type A aortic dissection and this represents a surgical emergency. Acute type A aortic dissection (ATAAD) is the leading cause of morbidity and mortality for patients with thoracic aortic aneurysms despite significant advances in the surgical treatment of aortic dissections. Prevention of ATAAD is challenging and includes optimal medical therapy for blood pressure and vascular health, as well as elective surgical proximal aorta (aortic root, ascending aorta) replacement in patients deemed at high risk for dissection^1–3^. The major risk factors for ATAAD are heritable genetic variants in genes predisposing to thoracic aortic disease, existence of a thoracic aortic aneurysm, male gender, hypertension, age and vascular risk factors^4–6^.

The investigators for the International Registry of Acute Aortic Dissection (IRAD) reported on 969 patients with acute aortic dissection from 17 cooperating centers around the globe and found a higher incidence of acute aortic dissections during winter months (28%) compared to summer months (20%). This association was independent of climate and temperature, and the observed seasonal variation suggests that other environmental factors may influence the risk for aortic dissection^7^.

We queried whether influenza could be such a seasonal factor and possibly associated with increased risk for ATAAD.

## Materials and Methods

This study was approved by the Institutional Review Board at the University of Michigan. We included all patients who presented to the University of Michigan and were diagnosed with ATAAD (defined as symptom onset ≤ 14 days of admission) between July 1996 and April 2019 (n = 758). Data was extracted from the University of Michigan Cardiac Surgery Data Warehouse. Detailed patient characteristics and surgical outcome of this cohort were previously reported by Yang and colleagues^8^. Our study does not include patient with type B aortic dissection since this group does not always undergoes surgical treatment.

All admissions for TAAD over a 23-year period between July 1996 and April 2019 were analyzed per calendar month January–December. Monthly ambient air temperature was gathered for Ann Arbor, Michigan between 1991–2019 from National Oceanic and Atmospheric administration (ncdc.noaa.gov) and we defined the winter season as “November–March” based on average low temperatures <35 F and compared to “April–October” with average low temperature >35 F.

Michigan-specific influenza trends are collected by the Michigan Department of Health and Human Services and were obtained for 2009–2019 from the Center for Disease Control and Prevention (CDC) website, including data from the Influenza-like Illness Surveillance Network (ILI-net), which reports percentage of patients who present to sentinel providers with flu-like symptoms, as well as from the Influenza Hospitalization Surveillance Project (IHSP), which reports on flu-related hospitalizations^9^. We also used surveillance data on lab confirmed cases of influenza provided by the University of Michigan Infectious Disease department (UMID) from 2013–2019 as an additional comparator group. Using the above sources, we calculated the average ILI, IHSP and UMID for each year (October–September) and then calculated monthly averages. We defined a month as having “high influenza activity” if the monthly average ILI, IHSP or UMID was above the corresponding yearly average. Conversely a month with “low influenza activity” was determined if ILI, IHSP and UMID average per month was below that of the yearly average. The number of monthly admissions for ATAADs during months with high influenza activity was compared to months with low influenza activity. Additionally, the association between monthly average rates from ILI, IHSP and UMID data and monthly admission for ATAADs was all independently assessed.

Statistical Analysis were performed using Microsoft Excel for statistical computing and SAS, version 9.4. Data are presented as mean (standard deviation). P-values are calculated using the student’s t-test, X-square or Fisher exact test for categorical data. Wilcoxon rank sum test was used for nonparametric comparison of continuous variables. A Poisson regression model was used to evaluate the association of influenza activity with monthly ATAAD admission rate, adjusting for monthly temperature. A rate ratio for aortic dissection in high influenza activity months versus low influenza activity months was derived from this model with inverse probability of treatment weighting (IPTW) by propensity score that included average monthly temperature as a covariate^10^. Statistical significance was set at p ≤ 0.05.

## Results

### Seasonal variability and association with regional influenza activity

**Figure 1:**
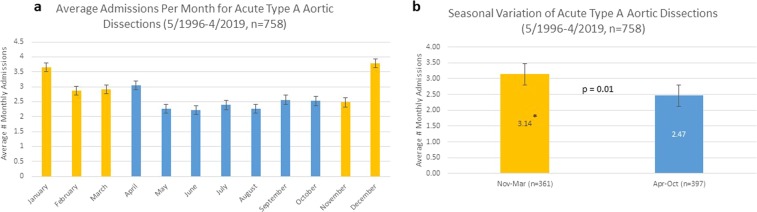
Average admission per calendar month (**a**) and per month stratified into winter season (November-March) and non-winter season (April-October) (**b**) for acute type A aortic dissection at the University of Michigan Medical Center between 7/1996 and 4/2019 (n = 758). In yellow are months with average ambient air temperature <35 F and <50 F for lowest and highest daily temperature, and in blue are months with average ambient air temperature >35 F and >50 F for lowest and highest daily temperature.

The average number of monthly admission for ATAAD to the University of Michigan Medical Center from July 1996–April 2019 is shown in Fig. 1a. We found a significantly higher average admission rate during the winter months November–March with 3.1 (SD 0.55) admission/months compared to 2.5 (SD 0.29) admission/month for the rest of the year (p = 0.01) as shown in Fig. 1b.

**Figure 2:**
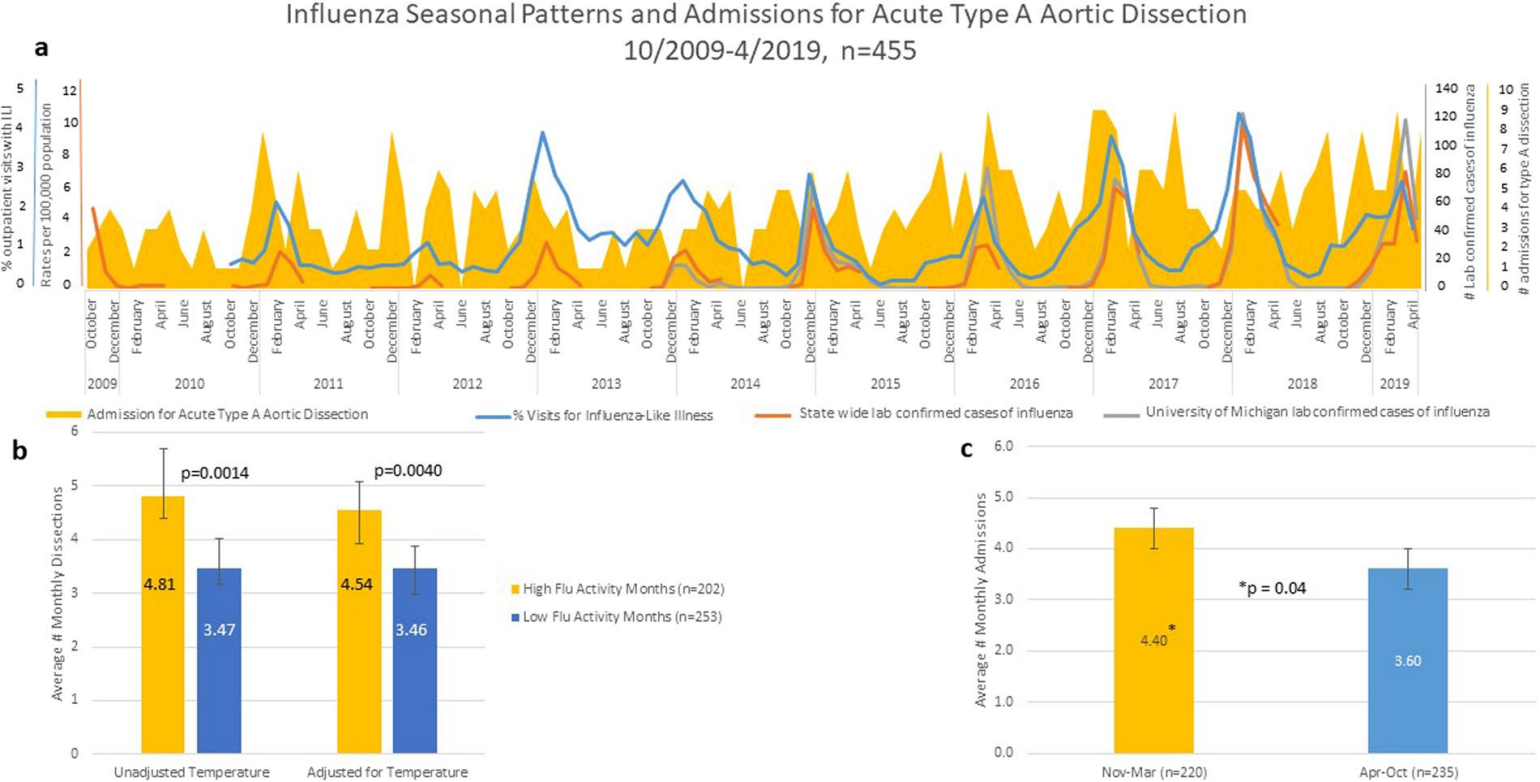
(**a**) Influenza patterns and average admissions per month for acute type A aortic dissection for all 455 patients admitted between 10/2009 and 4/2019. (**b**) Data stratified by influenza monthly activity determined by monthly rates of influenza-like illness, state lab confirmed influenza hospitalization and University of Michigan lab confirmed cases of influenza. Data both unadjusted and adjusted for temperature. (**c**) Data stratified by month between winter season (yellow, average temperature <35 F) and non-winter season (blue, average temperature >35 F).

Since statewide laboratory-confirmed hospitalizations for influenza became available starting October 2009, we overlaid monthly admission for ATAAD and influenza seasonal patterns for all 455 patients admitted with ATAAD between 10/2009–4/2019 as shown in Fig. 2a. Importantly, when ATAAD admissions per month were stratified according to influenza activity, there were significantly more admissions per month during those with high influenza activity compared to months with low influenza activity (4.8 and 3.5, respectively, p = 0.001, Fig. 2b). An IPTW Poisson regression model demonstrated that ATAAD admissions were still significantly higher during months with high influenza activity than those with low influenza activity after propensity score weighting using average monthly temperature as a covariate in the propensity score model (4.5 and 3.5, respectively) corresponding to an estimated rate ratio for monthly aortic dissections of 1.31 (95% CI: 1.09–1.58, P = 0.004) for peak influenza months vs. non-peak influenza. Similarly, ATAAD cases (October 2009–April 2019, n = 455) stratified according to ambient air temperature into winter months November-March (defined as average low air temperature <35 F), there were more admissions per month in winter months than non-winter months (4.4 and 3.6, respectively, p = 0.04, Fig. 2c). When comparing ATAAD admission rates with each individual marker of influenza activity, significantly higher monthly admission rates for ATAAD occurred during months with high rates of Influenza-like Illness (ILI, p = 0.02), state- confirmed influenza hospitalizations (IHSP, p = 0.05), but no significant association was found between ATAAD and UMID lab confirmed influenza (p = 0.1); Supplement Fig. 1.

Baseline characteristics of the entire cohort (n = 758) and the sub cohort (n = 455), starting from October 2009 when statewide data on influenza activity became available, is shown in Table 1. Average age of ATAAD patients was 58.6 years and 68.1% were male. There was no significant difference in patient demographics when stratified by admission during peak influenza months compared to non-peak influenza months, although there was a trend toward older and non-Marfan patients during the peak influenza months (Table 2).

**Table 1:** Baseline characteristics of patients with acute type A aortic dissection admitted to the University of Michigan Medical Center.

	Total Cohort (7/1996-4/2019)	Sub-cohort (10/2009-4/2019)	P-value
Number	758	455	—
Average Age (SD)	58.64 (13.42)	59.42 (13.14)	0.32
Male	68.74%	66.59%	0.44
Hypertension	74.35%	77.16%	0.27
Coronary Artery Disease	19.15%	16.62%	0.27
Diabetes	6.37%	7.32%	0.55
Marfan Syndrome	3.79%	2.43%	0.20

**Table 2:** Baseline characteristics of patients with acute type A aortic dissection admitted to the University of Michigan Medical Center between 10/2009 and 4/2019 (n = 455) and stratified by months with high and low influenza activity.

	High influenza activity months	Low influenza activity months	P-value
Number	202	253	—
Average Age (SD)	60.57 (13.19)	58.50 (13.06)	0.10
Male	67.66%	65.73%	0.66
Hypertension	80.00%	74.90%	0.20
Coronary Artery Disease	16.50%	16.73%	0.95
Diabetes	7.00%	7.57%	0.82
Marfan Syndrome	1.00%	3.58	0.076

### In-hospital and 30-day Mortality

Patient undergoing surgery for ATAAD between October 2009 and April 2019 were stratified into months with high influenza activity and low influenza activity. When comparing outcomes between the two groups, there was a significantly higher in-hospital mortality (10.99% vs. 5.79%, p = 0.024) and 30-day mortality (9.68% vs. 5.44%, p = 0.048) in patients presenting during months with high flu activity. There were no significant differences when comparing delay between admission and surgery (1.32 days v. 0.93 days, p = 0.13), length of admission (13.5 days vs. 13.9 days, p = 0.33) and emergent status (83.68% vs. 84.30%, p = 0.43). We also compared rates of post-operative pneumonia between high and low influenza activity months, and found no significant difference (16.23% vs. 15.70%, p = 0.44). There were only four total documented cases of pneumonia present at the time of presentation (Table 3).

**Table 3:** Crude clinical outcome of operative patients stratified by months with high and low influenza activity for acute type A aortic dissections admitted to the University of Michigan Medical Center between 10/2009 and 4/2019 (n = 433).

	High influenza activity months (n = 191)	Low influenza activity months (n = 242)	p-value
Surgery delay (days)	1.32	0.93	0.13
Length of Admission (days)	13.51	13.95	0.33
Post-Op Pneumonia	16.23%	15.70%	0.44
Emergent Status	83.68%	84.30%	0.43
In hospital Mortality	10.99%	5.79%	**0.024**
30-day mortality	9.68%	5.44%	**0.048**

## Discussion

Previous studies have shown seasonal differences in the occurrence of acute aortic dissections^7^, and a research abstract from the University of Texas at Houston reported on higher admission rates for ATAADs during winter months with a positive correlation with influenza activity based on statistics from the United States CDC^11^. Our data confirm those reports and show a statistically significant positive association with *regional* influenza activity and higher admission rates for ATAAD. While we cannot control for all environmental factors that occur during cold weather/winter months and influenza season, such as weather changes, behavioral changes, and possibly others, Poisson regression analysis adjusted for average monthly temperature shows a significant statistical association of monthly admissions for ATAAD at times of high regional influenza activity compared to low influenza season. This raises the hypothesis that influenza virus infection, but not the cold temperature itself, could be a triggering events subsequently resulting in acute aortic dissection. However, to establish causality, further studies with prospectively collected individual patient data and biological samples are needed to test whether influenza virus infection plays a role for ATAAD.

The mechanisms by which influenza, at least in susceptible patients, increases the risk for ATAAD is not clear since influenza viremia does not occur frequently in humans and the pathogenesis for cardiac complications of influenza is thought to be due to inflammatory and immune-mediated cardiac tissue injury, rather than direct influenza viral invasion of myocardial or other cardiac tissue^12,13^. However, systematic studies to test whether influenza RNA can be detected in human aortic tissue from patients with type A aortic dissection has not been performed to our knowledge. In contrast, experimental data obtained in mice with intranasal inoculation with H3N2 influenza virus in four different types of mice (ApoE null, LDLR null, C57BL6/J and outbred Swiss) was sufficient to detect influenza virus mRNA in aortic tissue (in 80% of mice) together with increased mRNA levels for MCP-1, IP-10, RANTES, IL-6, IL-1 and others genes consistent with a picture of influenza virus -mediated aortitis^14^. Authors of this study did not report on aortic dissection in these different mouse strains. Aortic inflammation and inflammasome-caspase 1-mediated degradation of vascular smooth muscle has been reported in human aortic tissue at the time of acute thoracic aortic dissections^15^. Moreover, our laboratory reported on S100A12, a pro-inflammatory and pro-apoptotic protein belonging to the class of damage associated molecular pattern molecules, to be highly expressed in the smooth muscle cells and invading myeloid cells in the dissecting aortic tissue harvested from patients with acute type A aortic dissection^16^. S100A12 is not endogenously expressed in healthy aorta, but is induced in a variety of cells in response to pathogens, cytokines, and cell stress and participates via activation of the Receptor for Advanced glycation Endproducts (RAGE) and toll-like receptor (TLR) in the regulation of inflammation and cell death^16^.

Clinically, it has not yet been shown whether the higher incidence of acute aortic dissections during influenza and winter season translates to worse intra- and post-operative outcomes, as one would suspect if indeed the aortic tissues would be more inflamed or friable from influenza virus. Our data show increased in-hospital mortality and 30-day mortality for ATAAD patients admitted during months with high influenza activity. The cause of increased mortality is unclear and not explained by postoperative pneumonia as shown in Table 3. It would be of considerable interest to know, whether patients vaccinated with the influenza vaccine have a lower risk for ATAAD or have less associated morbidity and mortality. Unfortunately, we have no reliable data on the influenza virus vaccination status for patients admitted with ATAAD to University of Michigan Medicine. Although the Michigan Care Improvement Registry collects data on vaccination for adults since 2006, the reporting by the providers of influenza vaccine to the Michigan Care Improvement Registry is not mandatory and hence incomplete.

A limitation of our study is that we do not have individual patient data on influenza virus infection to definitively address whether influenza virus infection affects the clinical outcome of surgically treated ATAAD patients, since the treating physician determines laboratory testing for respiratory viruses in ATAAD patients and there is no routine screening program in place. Therefore, we cannot definitely determine whether concomitant or recent influenza virus infection alters the clinical outcome in patients with ATAAD.

It is estimated that up to 50% of patients with acute aortic dissection die before reaching the hospital^17^ and it is conceivable that this could contribute to the increased incidence of cardiovascular mortality during peak winter months^18,19^. Most of the investigations has been geared towards the higher incidence of acute myocardial infarction (AMI) during peak winter months^20–22^, which has been associated with laboratory-confirmed influenza virus infection^12^. A role for influenza virus as a direct cause for AMI has been well documented^12,13,23–26^, and while a similar mechanism is plausible in ATAAD, it has yet to be proven. Importantly, there are studies to show decreased cardiovascular mortality in patients who have been vaccinated against influenza^27^, and as a result of those studies, the American Heart Association (AHA) and American College of Cardiology (ACC) give it a class I indication to provide influenza vaccination to all individuals with coronary and atherosclerotic vascular disease^28^. A clinical trial sponsored by the National Heart, Lung and Blood Institute to investigate whether high dose trivalent influenza vaccine will reduce cardiopulmonary events to a greater extent than standard dose quadrivalent influenza vaccine in 9300 high risk cardiovascular patients with a recent history of myocardial infarction or heart failure is ongoing (Clinical trials.gov identifier: NCT02787044).

Taken together, we report on a temporal association between regional influenza activity and increased admission for ATAAD, and further investigations are needed to better understand the mechanisms behind it. Increased expression of pro-inflammatory and pro-apoptotic pathways in the aorta induced by influenza virus or other co-circulating respiratory viruses is an attractive hypothesis, and needs to be tested in future studies by utilizing collection of a respiratory virus panel on patients admitted with ATAAD during months with above average influenza activity. However, other phenomena unrelated to influenza or other respiratory virus infections cannot be excluded. For example, it is possible that medication use such as antibiotics could account for more ATAAD during the influenza peak season. The U.S. Food and Drug administration recently warned that fluoroquinolone antibiotics can increase aortic dissections and should not be used in patients at increased risk unless there are no other treatment options available^29^.

A strength of our study is the use of the surgical database of the University of Michigan which collects data on every case of acute type A aortic dissection admitted to the hospital. A main limitation of our study is that we cannot exclude confounding factors present during peak influenza season, including but not limited to shoveling snow, less compliance with prescription refills and doctor visits and possibly poorly controlled blood pressure in the winter months. We propose that more research and awareness should be directed to better understand the association between possible influenza virus infection and increased admission rate for ATAAD.

## Electronic supplementary material

Supplementary Information.
