# Supplementary material for: Higher admission rates and in-hospital mortality for acute type A aortic dissection during Influenza season: a single center experience
Source: Sci Rep. 2020 Mar 13;10:4723. doi: 10.1038/s41598-020-61717-5 (PMC7070060; doi:10.1038/s41598-020-61717-5)
Supplement: Supplementary file 1 — Supplementary Information. [file 41598_2020_61717_MOESM1_ESM.pdf]

Higher admission rates and in-hospital mortality for acute type A aortic dissection during Influenza season: a single center experience

Carmel Ashur MD, Elizabeth Norton, Linda Farhat, Anna Conlon PhD, Cristen Willer PhD, James B. Froehlich MD, David J. Pinsky MD, Karen M. Kim MD, Shinichi Fukuhara MD, Michael G. Deeb, MD, Himanshu Patel MD, Kim A. Eagle MD, Yang Bo MD, Marion A. Hofmann Bowman MD

Supplement figure 1: Comparison of acute type A aortic dissection admission rates between months with high and low flu activity, as determined by Influenza Like Illness Network (a), Influenza Hospital Surveillance Project (b) and University of Michigan lab confirmed cases (c).

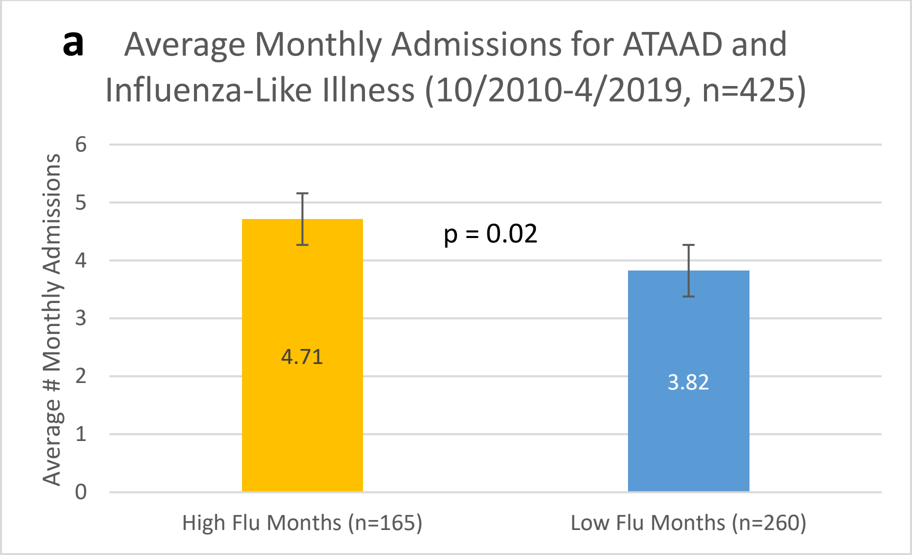

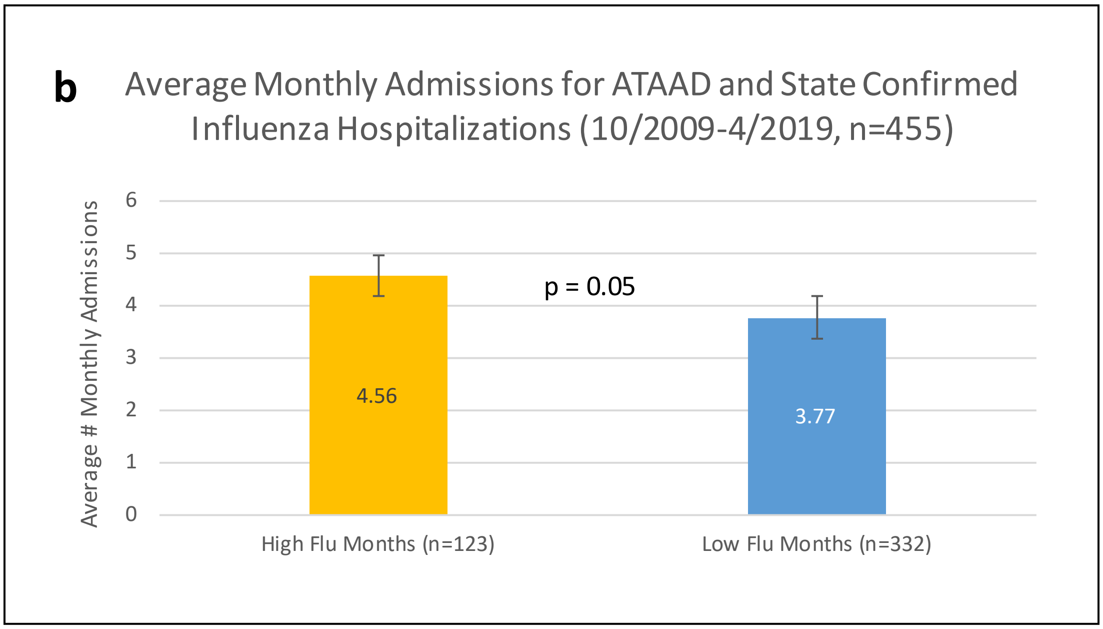
b
Average Monthly Admissions for ATAAD and State Confirmed Influenza Hospitalizations (10/2009-4/2019, n=455)
Average # Monthly Admissions
6
5
4
3
2
1
0
p = 0.05
4.56
3.77
High Flu Months (n=123)
Low Flu Months (n=332)

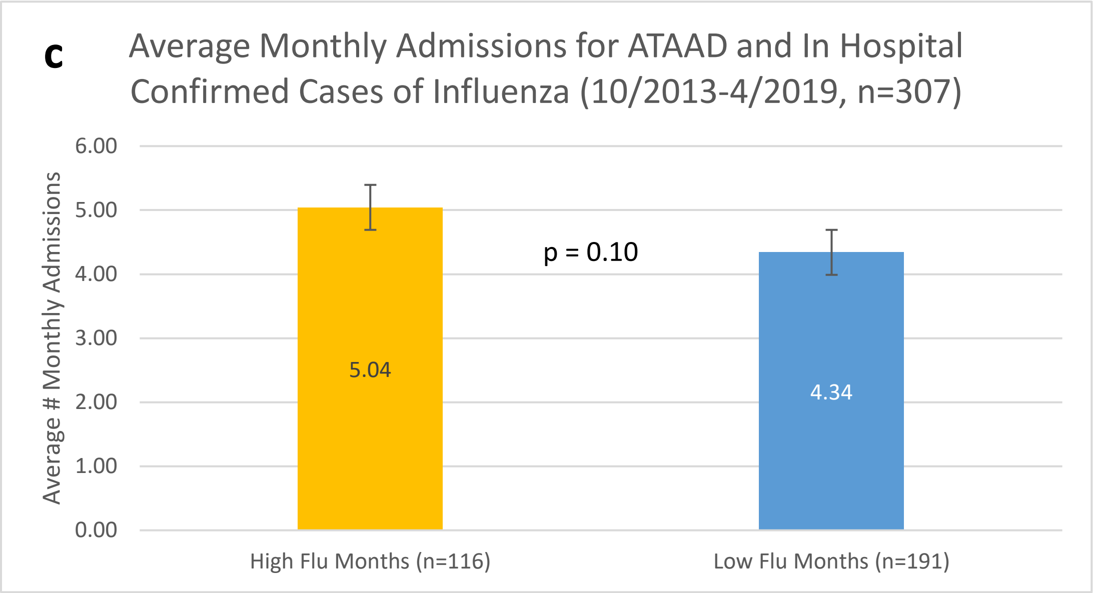
c
Average Monthly Admissions for ATAAD and In Hospital Confirmed Cases of Influenza (10/2013-4/2019, n=307)
Average # Monthly Admissions
6.00
5.00
4.00
3.00
2.00
1.00
0.00
p = 0.10
5.04
4.34
High Flu Months (n=116)
Low Flu Months (n=191)
